# Proteomic analysis of synaptic protein turnover in the anterior cingulate cortex after nerve injury

**DOI:** 10.1186/s13041-020-0564-y

**Published:** 2020-02-12

**Authors:** Hyoung-Gon Ko, Dong Ik Park, Ji Hyun Lee, Christoph W. Turck, Bong-Kiun Kaang

**Affiliations:** 1 Department of Anatomy and Neurobiology, School of Dentistry, Kyungpook National University, 2177 Dalgubeol-daero, Daegu, 41940 South Korea; 2 Department of Biological Sciences, College of Natural Sciences, Seoul National University, 1 Gwanangno, Gwanak-gu, Seoul, 08826 South Korea; 3 Department of Translational Research in Psychiatry, Max Planck Institute of Psychiatry, Kraepelinstr. 2, D-80804 Munich, Germany

## Abstract

Synaptic proteins play an important role for the regulation of synaptic plasticity. Numerous studies have identified and revealed individual synaptic protein functions using protein overexpression or deletion. In neuropathic pain nociceptive stimuli conveyed from the periphery repetitively stimulate neurons in the central nerve system, brain and spinal cord. Neuronal activities change the turnover (synthesis and degradation) rate of synaptic proteins. Thus, the analysis of synaptic protein turnover rather than just expression level change is critical for studying the role of synaptic proteins in synaptic plasticity. Here, we analyzed synaptosomal proteome in the anterior cingulate cortex (ACC) to identify protein turnover rate changes caused by peripheral nerve injury. Whereas PKCγ levels were not altered, we found that the protein’s turnover rate decreased after peripheral nerve injury. Our results suggest that postsynaptic PKCγ synthesized by neuronal activities in the ACC is translocated to the postsynaptic membrane with an extended half-life.

## Main text

External stimuli can change the efficacy of synaptic transmission, referred to as synaptic plasticity that underlies normal and pathophysiological brain functions including learning, emotion, cognition and pain. The study of synaptic protein function is critical for understanding how synaptic plasticity is regulated. The majority of studies on synaptic protein function have manipulated protein levels through deletion, reduction, and/or overexpression in cultured cells and/or animal models. In order to sustain synaptic function proteins have a finite life time that ranges from a few hours to months [1, 2] caused by continuous synthesis and degradation. Specific signals or stimuli can induce a change of synaptic protein turnover rate that contributes to synaptic plasticity [3–5].

Neuropathic pain is a disease known to be induced by abnormal signal intensification in parts of a pain signaling circuit such as spinal cord or supraspinal level without external injury. Many studies have shown that the anterior cingulate cortex (ACC) plays a role in the affective mode of pain including neuropathic pain [6, 7]. It is well known that in the ACC synaptic proteins contribute to the induction of neuropathic pain by regulating synaptic plasticity [8]. In previous study, we have analyzed turnover rate changes of synaptic proteins with a molecular weight greater than 90 kDa [9]. Using LC-MS analysis we found that NCAM1 has a rapid turnover in the ACC following peripheral nerve injury which plays an important role in long-term potentiation and neuropathic pain. In the current study, we have extended our investigation of a neuropathic pain mouse model to the turnover analysis of ACC synaptic proteins with a molecular weight smaller than 90 kDa.

**Fig. 1:**
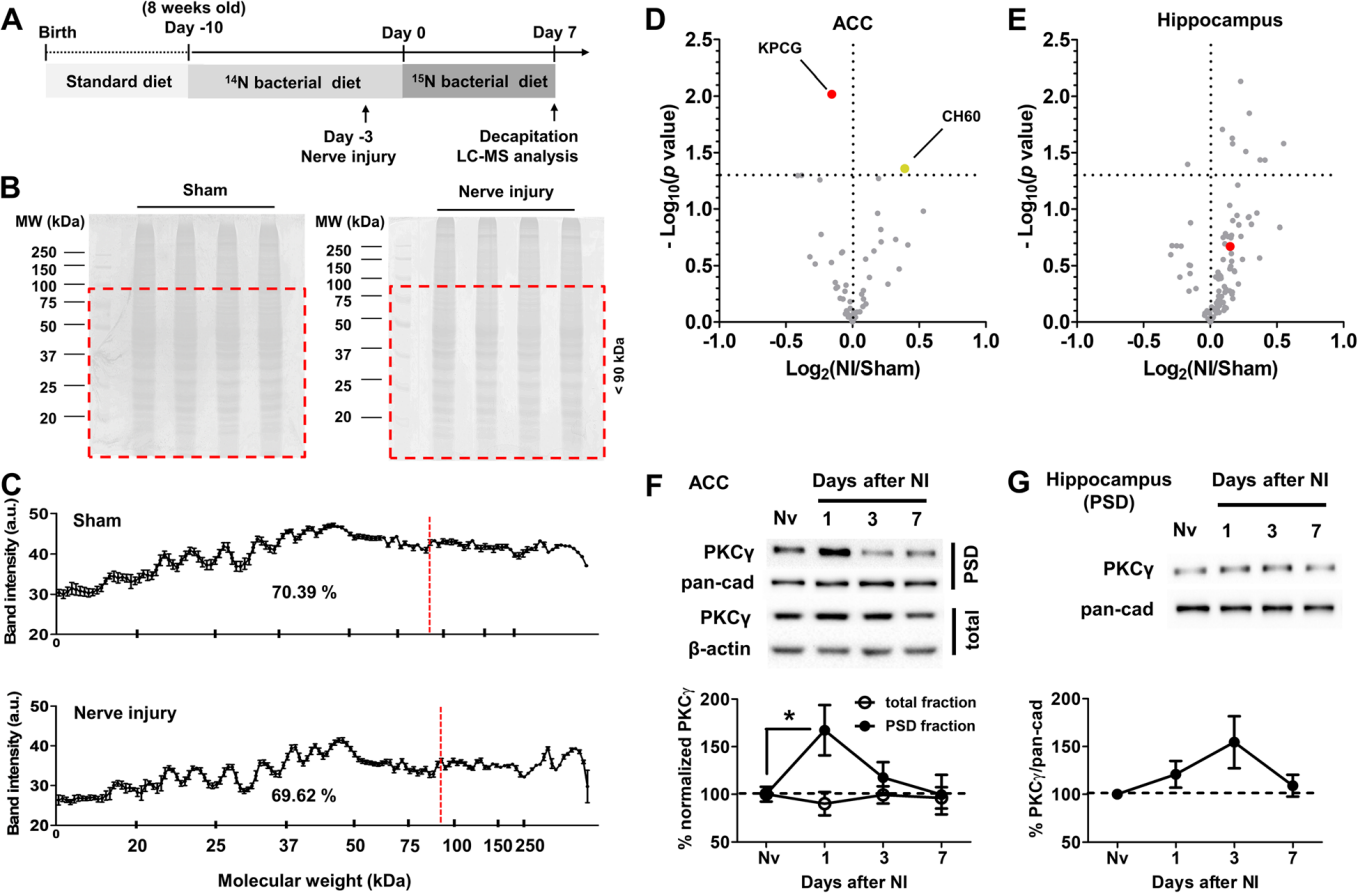
Turnover rate of postsynaptic PKCγ in the ACC slows down after peripheral nerve injury. **a** Experimental schedule of proteomic analysis for examining the change of protein turnover rate. For adaptation mice were fed with ^14^N food pellets for 1 week before CPN ligation. Subsequently the diet was switched to ^15^N food pellets. The ACC and hippocampus were dissected 1 week after ^15^N diet consumption and used for LC-MS analysis. **b** SDS electrophoresis gel images. Red dotted boxes indicate proteins smaller than 90 kDa. Left; Sham group, Right; Nerve injury group. **c** Densitometric analysis of Coomassie blue-stained gels. Red dot lines indicate smaller than 90 kDa. **d** and **e** Volcano plots of fold change of % labeled peptide fraction (LPF) for each protein in the ACC (**d**) and hippocampus (**e**). Proteins with -log10 (*p* value) > 1.301 were considered significant. (*n* = 3 or 4 mice per group, unpaired t-test). KPCG; protein kinase C gamma. CH60; mitochondrial 60 kDa heat shock protein (see also Additional file 1: Table S1). **f** PKCγ levels in total and PSD fraction of the ACC show a opposite change after peripheral nerve injury. Pan-cadherin (pan-cad) and β-actin were used for loading control. Upper; Representative western blot image, Lower; Quantitative analysis of Western blot image (PSD fraction, *n* = 12 per group, one-way ANOVA test followed by Bonferroni’s Multiple Comparison Test; F_(3,44)_ = 2.915, *p* < 0.05; posttest, * *p* < 0.05, total fraction, *n* = 10~11 per group, one-way ANOVA test; F_(3,37)_ = 2.098, *p* > 0.05). **g** PKCγ levels in the PSD fraction of the hippocampus do not show a significant alteration. Pan-cadherin was used for loading control. Upper; Representative Western blot image, Lower; Quantitative analysis of Western blot image (*n* = 10 per group, one-way ANOVA test; F_(3,36)_ = 2.126, *p* > 0.05)

To examine synaptic protein turnover rates we used a partial stable isotope metabolic labeling method (Fig. 1a). Briefly, 8 weeks old male mice were first fed with a ^14^N diet for 10 days for food pellet adaptation. Following ligation of the common peroneal nerve (CPN) with a wax coated braided suture the animals were switched to a ^15^N bacterial diet. After 7 days, mice were decapitated and the crude ACC synaptosomal P2 fraction was purified for LC-MS analysis. SDS polyacrylamide gel electrophoresis showed that approximately 70% of all synaptic proteins had a molecular weight of less than 90 kDa with no significant expression level difference between sham and nerve injury group (Fig. 1b and c; Sham = 70.39 ± 0.15%, Nerve injury = 69.62 ± 0.36%, unpaired t-test, *p* > 0.05). We therefore decided to analyze turnover rather than expression level differences of these proteins induced by peripheral nerve injury. Following in-gel tryptic digestion and peptide extraction we calculated the ^15^N-labeled peptide fraction (LPF) using the in-house developed ProTurnyzer software [10]. The NI/Sham LPF ratio of two ACC proteins, KPCG (protein kinase C gamma, PKCγ) and CH60 (mitochondrial 60 kDa heat shock protein, HSP60) indicated a significant turnover rate change following peripheral nerve injury (Fig. 1d and Additional file 1: Table S1; Sham vs. Nerve injury, unpaired t-test, PKCγ, *p* < 0.01, HSP60, *p* < 0.05). While CH60 has been shown to be one of the components in proteolytic machinery at synapse and thus may play a role in protein turnover regulation [11], mitochondrial contamination in the synaptosome preparation cannot be excluded. Therefore, our subsequent analysis focused on PKCγ. When we analyzed the hippocampus, we did not observe any significant PKCγ turnover change between sham and nerve injury groups (Fig. 1e, Sham vs. Nerve injury, unpaired t-test, PKCγ, *p* > 0.05). This result shows a region-specific change of PKCγ turnover rate after peripheral nerve injury. We next examined PKCγ level changes in the ACC after peripheral nerve injury using Western blot. The ACC tissue was dissected from naïve and nerve injury-operated mice 1, 3 or 7 days after surgery and PSD fraction prepared by sucrose gradient centrifugation. PKCγ levels in PSD fraction of the ACC showed a significant change over time (Fig. 1f; one-way ANOVA followed by Bonferroni’s Multiple Comparison Test; F_(3,44)_ = 2.915, *p* < 0.05; posttest, * *p* < 0.05) showing enhancement 1 day after nerve injury and then returning to basal level. However, total PKCγ showed no significant change over time. Also, no significant changes were found in PSD fraction of the hippocampus (Fig. 1g; one-way ANOVA test; F_(3,36)_ = 2.126, *p* > 0.05).

In this study, we assessed the turnover rate of ACC synaptic proteins smaller than 90 kDa and identified PKCγ that showed a smaller turnover rate after peripheral nerve injury. The PKCγ isoform has the unique feature of neuron specific expression where it is localized in the soma, dendrite and axon. In the synapse, PKCγ is exclusively located in the postsynaptic site (dendritic spine), but not in the presynaptic terminal [12, 13]. Interestingly, PKCγ translocates between the cytoplasm and membrane depending on the specific stimulus [14, 15]. These characteristics suggest that PKCγ is involved in synaptic plasticity. In fact, a previous study using PKCγ knock out (KO) mice reported that PKCγ is required for LTP in the hippocampus [16]. Also, it is well known that in the ACC LTP is involved in the cellular mechanism of neuropathic pain. Our results show that synaptic PKCγ levels in the ACC quickly increase and subsequently return to basal levels by slowing down its turnover rate after peripheral nerve injury. Since total PKCγ levels did not change, these results imply that PKCγ may be involved in the formation of neuropathic pain by translocating rapidly to the specific postsynaptic membrane activated by neuronal stimuli induced by peripheral nerve injury, but gradually reduced in postsynapse which is not associated with pain processing. In addition, translocated synaptic PKCγ may perform stable functions in the synaptic region with extended half-life.

The present study is the first one that examines the brain region-specific role of PKCγ in neuropathic pain. PKCγ-deficient mice show a diminished neuropathic pain phenotype after peripheral nerve injury [17]. In the spinal cord, PKCγ interneurons receive Aβ afferent input and contribute to the transformation of tactile to nociceptive information as an excitatory interneuron [18]. While multiple studies have investigated the role of spinal PKCγ, the protein has been rarely studied in relation to neuropathic pain in other brain regions. Future brain region-specific PKCγ deletion or knockdown mice will help to further elucidate the novel role of PKCγ in neuropathic pain.

## Supplementary information

**Additional file 1: Table S1.** The LPF profiles of synaptic proteins smaller than 90 kDa in the ACC and hippocampus of the sham and nerve-injured mice. Red indicates *p* < 0.05 in unpaired t-test. The hippocampal P2 fraction was examined as a negative control.

## Data Availability

All data analyzed in this study were included in this article. The detailed material and method information is presented in our previous study [9].

## References

[1] Li KW, Hornshaw MP, Van Der Schors RC, Watson R, Tate S, Casetta B (2004). Proteomics analysis of rat brain postsynaptic density. Implications of the diverse protein functional groups for the integration of synaptic physiology. J Biol Chem.

[2] Peng J, Kim MJ, Cheng D, Duong DM, Gygi SP, Sheng M (2004). Semiquantitative proteomic analysis of rat forebrain postsynaptic density fractions by mass spectrometry. J Biol Chem.

[3] Alvarez-Castelao B, Schuman EM (2015). The regulation of synaptic protein turnover. J Biol Chem.

[4] Choi JH, Kim JE, Kaang BK (2010). Protein synthesis and degradation are required for the incorporation of modified information into the pre-existing object-location memory. Mol Brain.

[5] Kaang BK, Lee SH, Kim H (2009). Synaptic protein degradation as a mechanism in memory reorganization. Neuroscientist.

[6] Bliss TV, Collingridge GL, Kaang BK, Zhuo M (2016). Synaptic plasticity in the anterior cingulate cortex in acute and chronic pain. Nat Rev Neurosci.

[7] Kang SJ, Kwak C, Lee J, Sim SE, Shim J, Choi T (2015). Bidirectional modulation of hyperalgesia via the specific control of excitatory and inhibitory neuronal activity in the ACC. Mol Brain.

[8] Zhuo M (2009). Plasticity of NMDA receptor NR2B subunit in memory and chronic pain. Mol Brain.

[9] Ko HG, Choi JH, Park DI, Kang SJ, Lim CS, Sim SE (2018). Rapid turnover of cortical NCAM1 regulates synaptic reorganization after peripheral nerve injury. Cell Rep.

[10] Zhang Y, Reckow S, Webhofer C, Boehme M, Gormanns P, Egge-Jacobsen WM (2011). Proteome scale turnover analysis in live animals using stable isotope metabolic labeling. Anal Chem.

[11] Gorenberg EL, Chandra SS (2017). The role of co-chaperones in synaptic Proteostasis and neurodegenerative disease. Front Neurosci.

[12] Kose A, Ito A, Saito N, Tanaka C (1990). Electron microscopic localization of gamma- and beta II-subspecies of protein kinase C in rat hippocampus. Brain Res.

[13] Saito N, Shirai Y (2002). Protein kinase C gamma (PKC gamma): function of neuron specific isotype. J Biochem.

[14] Narita M, Mizoguchi H, Narita M, Nagase H, Suzuki T, Tseng LF (2001). Involvement of spinal protein kinase Cgamma in the attenuation of opioid mu-receptor-mediated G-protein activation after chronic intrathecal administration of [D-Ala2,N-MePhe4,Gly-Ol(5)]enkephalin. J Neurosci.

[15] Narita M, Aoki T, Ozaki S, Yajima Y, Suzuki T (2001). Involvement of protein kinase Cgamma isoform in morphine-induced reinforcing effects. Neuroscience.

[16] Abeliovich A, Chen C, Goda Y, Silva AJ, Stevens CF, Tonegawa S (1993). Modified hippocampal long-term potentiation in PKC gamma-mutant mice. Cell.

[17] Malmberg AB, Chen C, Tonegawa S, Basbaum AI (1997). Preserved acute pain and reduced neuropathic pain in mice lacking PKCgamma. Science.

[18] Miraucourt LS, Dallel R, Voisin DL (2007). Glycine inhibitory dysfunction turns touch into pain through PKCgamma interneurons. PLoS One.

